# Qian Yang Yu Yin Granule-containing serum inhibits angiotensin II-induced proliferation, reactive oxygen species production, and inflammation in human mesangial cells via an NADPH oxidase 4-dependent pathway

**DOI:** 10.1186/s12906-015-0619-2

**Published:** 2015-03-25

**Authors:** Kang Ding, Yan Wang, Weimin Jiang, Yu Zhang, Hongping Yin, Zhuyuan Fang

**Affiliations:** First College of Clinical Medicine, Nanjing University of Traditional Chinese Medicine, Nanjing, 210046 China; Nanjing Hospital of Traditional Chinese Medicine, Nanjing, 210001 China; Jiangsu Province Hospital of Traditional Chinese Medicine, No.155 Hanzhong Road, Nanjing, 210029 China; School of Life Science and Technology, China Pharmaceutical University, Nanjing, 210009 China; College of Pharmacy, Nanjing University of Traditional Chinese Medicine, Nanjing, 210029 China

**Keywords:** Angiotensin II, NADPH oxidase, Reactive oxygen species, Human mesangial cells, Qian Yang Yu Yin Granule

## Abstract

**Background:**

Qian Yang Yu Yin Granule (QYYYG), a traditional Chinese herbal medicine, has been indicated for renal damage in hypertension for decades in China, but little remains known regarding its underlying molecular mechanism. Therefore, we performed the current study in order to investigate the underlying molecular mechanism of QYYYG in the treatment of hypertensive renal damage.

**Methods:**

We hypothesize that QYYYG relieves hypertensive renal injury through an angiotensin II (Ang II)-nicotinamide adenine dinucleotide phosphate (NAPDH)-oxidase (NOX)-reactive oxygen species (ROS) pathway. In this study, we investigated the effects of QYYYG-containing serum (QYGS) in human mesangial cells (HMCs) against Ang II-induced cell proliferation, ROS production, and inflammation through the seropharmacological method.

**Results:**

We found that QYGS could inhibit cell proliferation in Ang II-treated HMCs. In addition, QYGS considerably suppressed production of ROS, decreased mRNA and protein expression of NAPDH-oxidase 4 (NOX4), p22^*phox*^, and activated Ras-related C3 botulinum toxin substrate 1 (GTP-Rac1); as well as counteracted the up-regulation of inflammatory markers including tumor necrosis factor-α (TNF-α), nuclear factor-κB (NF-κB) p65, and interleukin 6 (IL-6). These effects were further confirmed in HMCs transfected with specific small interfering RNA (siRNA) targeting NOX4.

**Conclusions:**

Taken together, these results suggest that a NOX4-dependent pathway plays an important role in regulating the inhibitory effect of QYGS. Our findings provide new insights into the molecular mechanisms of QYYYG and their role in the treatment of hypertensive nephropathy.

## Background

Hypertension is one of the most common risk factors for cardiovascular and renal diseases [1]. Recently, it has been widely accepted that the renin-angiotensin system (RAS) plays an essential role in the pathophysiology of hypertension-related renal damage [2]. Numerous studies have demonstrated that angiotensin II (Ang II), the central effector molecule of the RAS, significantly contributes to the induction of irreversible renal damage through the direct or indirect stimulation of glomerular mesangial expansion, glomerular basement membrane reduplication, and extracellular matrix (ECM) deposition [3]. In addition, researchers have found that Ang II is able to induce contraction of glomerular mesangial cells, which contributes to the development of glomerular atrophy and sclerosis, resulting in progressive renal dysfunction [4,5]. Therefore, fully understanding the underlying molecular mechanism of Ang II-induced glomerular pathological changes has important implications for the prevention and treatment of renal damage in hypertension.

In recent years, a considerable number of studies have identified several specific signaling pathways that mediate the pathogenic actions of Ang II, of which a nicotinamide adenine dinucleotide phosphate (NADPH)-oxidase (NOX)-dependent pathway plays a pivotal role [6] Previous investigations have confirmed that Ang II induces reactive oxygen species (ROS) generation through the NOX system [5,7]. Then, oxidative stress from the ROS stimulates ROS-sensitive inflammatory pathways, such as nuclear factor-κB (NF-κB) cascade, to produce a number of pro-inflammatory mediators and cytokines [8,9]. These pro-inflammatory factors, together with Ang II-induced oxidative stress, are able to cause renal vascular remodeling and injury to the endothelia cells of renal small arteries. These effects decrease renal blood supply, ultimately leading to ischemic renal parenchymal injury [10]. Hence, the blockade of Ang II has been considered a major therapeutic strategy for hypertensive nephropathy.

Angiotensin receptor blockers (ARBs), the most widely-used anti-hypertensive drugs, have been recommended for use in the management of hypertensive nephropathy by the 2013 European guidelines for the management of arterial hypertension [11]. Numerous recent studies also have demonstrated the protective effects of ARBs against Ang II-induced glomerular pathological changes through blocking oxidative stress and oxidative stress-mediated inflammatory responses [12-14]. However, due to the pathogenic complexity of hypertensive nephropathy, there is still an increasing demand for novel and effective therapeutics.

In recent years, traditional Chinese medicine has shown its advantage against hypertension and hypertension-related complications [15]. Qian Yang Yu Yin Granule (QYYYG), a traditional Chinese herbal medicine, has been indicated for renal damage in hypertension for decades in China, but little remains known regarding its underlying molecular mechanism. *Polygoni Multiflori Radix*, one of the major ingredients of QYYYG, has been found to induce potent antioxidant and anti-inflammatory effects [16,17]. Therefore, we hypothesize that QYYYG relieves hypertensive renal injury through an Ang II-NOX-ROS pathway. In the present study, we investigated the effects of QYYYG-containing serum (QYGS) in human mesangial cells (HMCs) against Ang II-induced cell proliferation, ROS production, and inflammation through the seropharmacological method. We found that these effects of QYGS are likely to be mediated through a NOX4-dependent pathway, which may possibly be a molecular mechanism behind the pharmacological effects of QYYYG.

## Methods

### Reagents

QYYYG (batch No.1307004) was provided by the Institute of Chinese Traditional Medicine of Jiangsu Province (Nanjing, China). Detailed information on the components of QYYYG and their amounts is listed in Table 1. The preparation process of QYYYG was as follows: All components were mixed in proportion. The mixture was decocted with 10-fold amount of water (volume/weight) twice, for 1 h each time. The resultant filtrates were combined and concentrated to a relative density of 1.2 g/ml (at 60°C). After adding stevioside to a concentration of 1% (weight/volume), the concentrated filtrate was mixed with dextrin at a weight ratio of 1:1 and granulated in a fluidized bed granulator. The resulting granules were dried at 60°C for 24 h and then stored at 4°C for future use.

**Table 1:** **The components of QYYYG**

**Components**	**Chinese name**	**Origin**	**Amount used (g)**
Polygoni Multiflori Radix	He Shou Wu	*Polygonum multiflorum* Thunb.	180
Herba Bidentis Bipinnatae	Gui Zhen Cao	*Bidens pilosa Linnaeus*	110
Corni Fructus	Shan Zhu Yu	*Cornus officinalis*	108
Scrophulariae Radix	Xuan Shen	*Scrophularia ningpoensis*	180
Alismatis Rhizoma	Ze Xie	*Alisma orientalis*	180
Cyathulae Radix	Chuan Niu Xi	*Cyathula officinalis*	170

According to the Pharmacopoeia of the People’s Republic of China (2010 edition) and the Quality Specifications of Chinese Traditional Medicine of Jiangsu Province, thin-layer chromatography (TLC) and high performance liquid chromatogram (HPLC) methods were employed to control the quality of QYYYG (Table 2). Valsartan (Diovan®, batch No. X1584) was provided by Norvatis (Basel, Switzerland).

**Table 2:** **Quality evaluation of QYYYG**

**Major constituents**	**Method of determination**	**Quality specifications**
2,3,5,4′-tetrahydroxystilbene-2-O-β-D-glucopyranoside	HPLC	*>6 mg per 10 g QYYYG*
Polygoni Multiflori Radix	TLC	*Contained*
Herba Bidentis Bipinnatae	TLC	*Contained*
Corni Fructus	TLC	*Contained*
Cyathulae Radix	TLC	*Contained*

HPLC: high performance liquid chromatogram, TLC: thin-layer chromatography.

Ang II was purchased from Sigma (St. Louis, MO, USA). Dulbecco’s modified Eagle’s medium (DMEM), trypsin, and fetal bovine serum (FBS) were from Life Technologies (Carlsbad, CA, USA). Dimethyl sulfoxide (DMSO) and 3-(4,5-dimethyl-2-thiazolyl)-2,5-diphenyl tetrazolium bromide (MTT) were purchased from Amresco (Solon, OH, USA). TRNzol® Plus reagent was from Biouniquer (Nanjing, China).

### Cell culture

HMCs were provided by the Institute of Life Science and Technology, China Pharmaceutical University (Nanjing, China). The cells were cultured in DMEM supplemented with 10% FBS, 2 mM glutamine, 100 U/ml penicillin, and 100 mg/ml streptomycin at 37°C in a 5% CO_2_ atmosphere.

### Preparation of QYYYG-containing serum

Thirty Wistar rats (age of 14 weeks, weighing between 260 and 320 g) were provided by the Experimental Animal Center of Yangzhou (Yangzhou, China). The animal experiments were conducted in accordance with the guidelines as set forth by the National Research Council. The study protocol was approved by the Animal Ethics Committee of Nanjing University of Traditional Chinese Medicine.

All animals were maintained under temperature-controlled (22 ± 2°C) and humidity-controlled (55 ± 5%) conditions in 12-hour light/dark cycles with free access to sterile normal food and water. After an acclimation period of one week, the animals were randomly divided into three groups: the QYGS group, the valsartan-containing serum (VS) group, and the control serum group (n = 10 animals per group). Animals in the QYGS group and VS group were orally administered 2.5 g/kg of QYYYG (10-fold clinic dosage) and 13.3 mg/kg of valsartan (10-fold of clinic dosage) once daily for 7 days. Both drugs were suspended in 3 ml of distilled water. One hour after the last administration of QYYYG or valsartan on the seventh day, whole blood was obtained from the retro-orbital venous plexus of the animals in all groups. For all groups, serum was isolated by centrifugation at 3000 rpm for 10 min after standing at room temperature (RT) for 30 min, and then mixed with serum from the same group. Then, the serum was inactivated in a 56°C environment for 30 min and filtered with 0.22 μm pore-size membrane. The filtered serum was aliquoted and stored at −20°C for future use.

### Cell proliferation assay

MTT assay was used to evaluate the proliferation ability of Ang II-treated HMCs. Cells were transferred onto 96-well plates containing DMEM supplemented with 10% FBS. The optimal cell number was found to be 1 × 10^4^ cells/well. Cells were then treated with varying concentrations of Ang II (0, 10^−3^, 10^−4^, 10^−5^, 10^−6^, and 10^−7^ mmol/L). After 24 h, 20 μl MTT (5 mg/ml) was added and cells were cultured for an additional 4 h. Subsequently, the medium was removed and 150 μl DMSO was added to each well. After 5 min of shaking at RT, the absorbance was measured at 570 nm by a microplate reader (BioTek Instruments, Winooski, VT, USA). Assays were performed in sextuplicate and repeated four times independently. Based on the results, 10^−4^ mmol/L of Ang II was determined to be the optimal treatment concentration for subsequent studies.

MTT assay was also used to examine the inhibitory effects of QYGS on proliferation in Ang II-treated HMCs. The cells were cultured as described above, and then were pre-treated with VS or QYGS in groups as follows: the vehicle group (with medium containing 5% drug-free rat serum), the Ang II model group (with medium containing 5% drug-free rat serum), the 5% VS group (positive control, with medium containing 5% VS), the 1.25% QYGS group (with medium containing 1.25% QYGS and 3.75% drug-free rat serum), the 2.5% QYGS group (with medium containing 2.5% QYGS and 2.5% drug-free rat serum), and the 5% QYGS group (with medium containing 5% QYGS). After one hour, all groups except for the vehicle group were incubated with 10^−4^ mmol/L of Ang II for 24 h. Then, MTT assay was performed to detect cell proliferation in each group. Assays were performed in sextuplicate and repeated four times independently.

### Real time-polymerase chain reaction analysis

HMCs were cultured in 6-well plates at a density of 2 × 10^5^ cells per well. Then, the cells were grouped and pre-treated with VS or QYGS as described in the above section. One hour later, all groups except for the vehicle group were treated with 10^−4^ mmol/L of Ang II for 1 h. Afterwards, total RNA was extracted from cells using TRIzol Plus reagent (Biouniquer) according to the manufacturer’s instructions. RNA concentrations were determined by a microplate reader (BioTek Instruments). Total cDNA was synthesized with a cDNA synthesis kit (Biouniquer). RT-PCR was performed using an ABI 7700 Prism Sequence Detection System and TaqMan primer probes (Applied Biosystems, Foster City, CA, USA). The primer sequences are presented in Table 3. The total reaction volume was 20 μl: 5 μl cDNA, 10 μl 2 × UltraSYBR Mixture (with ROX), 0.5 μl of each primer, and 4 μl RNase-free water. Cycle parameters were as follows: activation at 95°C for 10 min, 40 cycles of denaturation at 95°C for 15 s, and then annealing and extension at 60°C for 60 s. Calculations of the expression levels were carried out using the absolute standard curve method [18]. Glyceraldehyde-3-phosphate dehydrogenase (GAPDH) was used as an internal control. All experiments were conducted three times independently.

**Table 3:** **Primers used in RT-PCR**

**Target gene**	**Sequence of forward and reverse primers**
NOX4	5′-TAGATACCCACCCTCCCG-3′
	5′-TGACTGGCTTATTGCTCC-3′
p22^*phox*^	5′- GCGGCATCTACCTACTGGC-3′
	5′- CTCCTCGCTGGGCTTCTT-3′
Rac1	5′-GAAGCAGCAGAAATCACAG-3′
	5′-CCAATACTCCAGAGGCAAG-3′
GTP-Rac1	5′-TTAGGGATGATAAAGACACG-3′
	5′-GGACAGGACCAAGAACGAG-3′
GAPDH	5′- GGATTTGGTCGTATTGGG-3′
	5′- GGAAGATGGTGATGGGATT −3′

### Western blot analysis

HMCs were cultured, grouped, and treated as described in the “Real time-polymerase chain reaction analysis” section. HMCs of all groups were then harvested by centrifugation at 1500 rpm for 5 min. The harvested cells were washed three times with phosphate-buffered saline (PBS, pH 7.2 – 7.4) and lysed in RIPA buffer by shaking at 4°C for 30 min. The homogenates were centrifuged at 14,000 rpm for 10 min and the supernatant was used for western blot analysis. Protein concentrations were determined using the Bradford method according to the manufacturer’s instructions (Beyotime Technology, Jiangsu, China). Supernatants were mixed with sodium dodecyl sulfate-polyacrylamidegel electrophoresis (SDS-PAGE) sample buffer and boiled for 10 min, and were then separated on 10% SDS–PAGE gel. Subsequently, samples were transferred onto polyvinylidene difluoride (PVDF) membranes (Bio-Rad, Hercules, CA, USA). Membranes were blocked for 1 h in 5% (w/v) non-fat milk and then incubated with a primary antibody at RT for 2 h. The following primary antibodies were used: NOX4, p22^*phox*^, Rac1, tumor necrosis factor-α (TNF-α), NF-κB p65, and interleukin 6 (IL-6) (Santa Cruz Biotechnology, Santa Cruz, CA, USA); activated Ras-related C3 botulinum toxin substrate 1 (GTP-Rac1) (NewEast Bioscience, Malvern, PA, USA); and β-actin (Sino Biological Inc., Beijing, China). Following incubation, the membranes were washed three times in TBST buffer, and then incubated with horseradish peroxidase (HRP)-conjugated rabbit IgG (diluted 1:5000) for 1 h at RT. After three washes with TBST, proteins were detected using an enhanced chemiluminescence (ECL) detection kit (Beyotime). The immunoblotted bands were quantified by Gel-Pro Analyzer software (version 4.0, Media Cybernetics, Rockville, MD, USA), and the protein of interest was normalized to β-actin. All experiments were performed three times independently.

### Reactive oxygen species generation

HMCs were cultured, grouped, and treated as described in the “Real time-polymerase chain reaction analysis” section. Then, 2′,7′-dichlorodihydrofluorescein diacetate (DCHF-DA) (Beyotime) was added to each culture at a final concentration of 10 μmol/L and incubated for 20 min at 37°C. The cells were washed with PBS three times and cellular fluorescence was assayed using cell flow cytometry (BD Bioscience, Bedford, MA, USA). Assays were repeated three times independently.

### Small interfering RNA against NOX4

Twenty-four hours prior to transfection, 2 × 10^5^ HMCs were seeded into 6-well plates and cultured in antibiotic-free medium to obtain 50-80% confluence. Then, transfection of 125 pmol small interfering RNA (siRNA) against NOX4 was performed using Lipofectamine 2000 (Life Technologies) according to the manufacturer’s instructions. The transfected HMCs were grouped and treated the same way as described previously. A nonspecific scrambled siRNA was used as a negative control (the RNAcon group). Cells from all groups were subjected to RT-PCR analysis, Western blot, and ROS detection assay.

### Statistical analysis

All statistical analysis was performed using SPSS 17.0 software (SPSS Inc., Chicago, IL, USA). Data are presented as means ± standard error of the mean (SEM). Differences between experimental groups are assessed for statistical significance using one-way analysis of variance (ANOVA), followed by least significant difference (LSD) or Games-Howell post-hoc multiple comparison tests for equal or unequal variances, respectively. A *P* value less than 0.05 was considered statistically significant (two-tailed).

## Results

### Effects of Ang II on proliferation of HMGs

**Figure 1:**
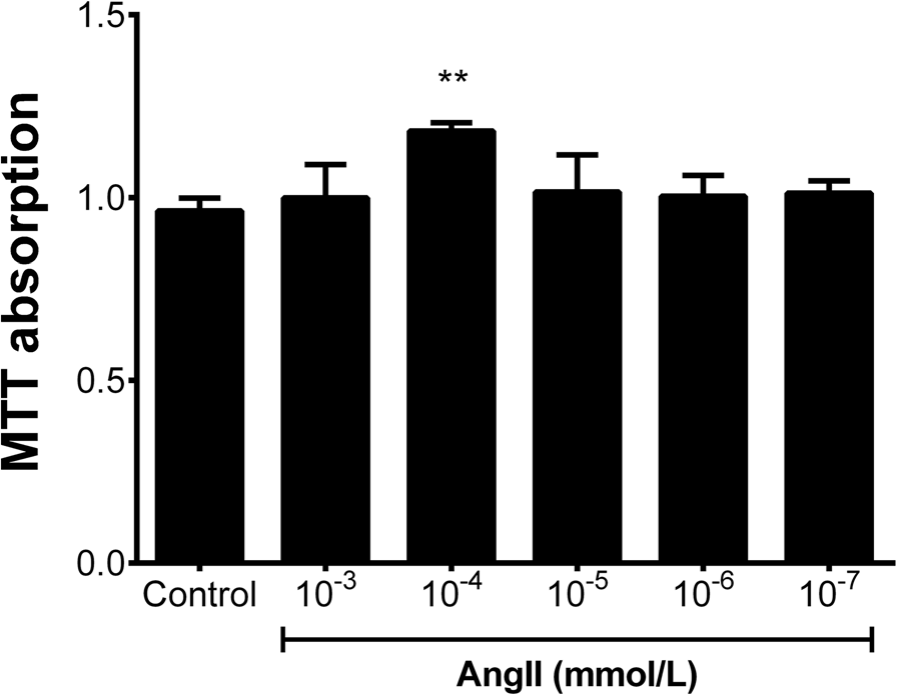
**Effects of Ang II on HMC proliferation after 24 h-treatment.** Cell proliferation was assessed using the MTT method. Data are presented as means ± SEM (n = 4). **P < 0.01 compared with the control group.

The effects of Ang II on proliferation of HMCs are shown in Figure 1. We found that only 10^−4^ mmol/L of Ang II could significantly promote the proliferation of HMCs when the cells were treated with various doses of Ang II (10^−3^ to 10^−7^ mmol/L in a 10-fold concentration gradient) for 24 h. Therefore, 10^−4^ mmol/L of Ang II was used in subsequent experiments.

### Inhibitory effects of QYGS on Ang II-induced HMC proliferation

**Figure 2:**
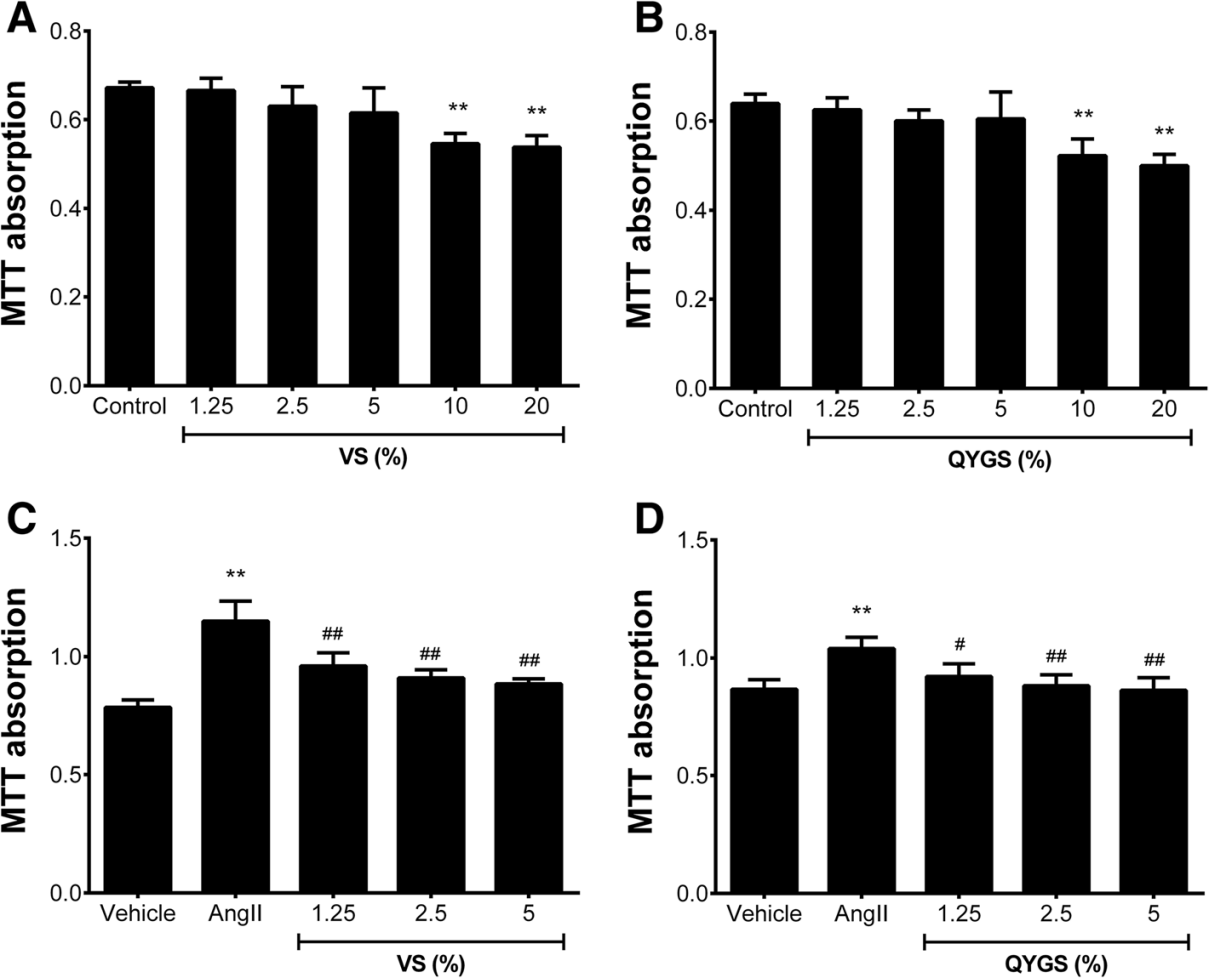
**Effects of QYGS on Ang II-induced HMC proliferation assessed by MTT assay. (A)** Effect of VS (0, 1.25%, 2.5%, 5%, 10%, and 20%) on cell proliferation after 24 h-incubation. **(B)** Effect of QYGS (0, 1.25%, 2.5%, 5%, 10%, and 20%) on HMC proliferation following incubation for 24 h. **(C)** and **(D)** Inhibitory effects of varying doses of VS or QYGS (1.25%, 2.5%, and 5%) on cell proliferation in Ang II-treated HMCs. Data are presented as means ± SEM (n = 4). **P < 0.01 as compared with the control group or the vehicle group. ^#^ *P* < 0.05, ^##^ *P* < 0.01 as compared with the Ang II model group.

Figure 2 presents the inhibitory effects of QYGS on Ang II-induced HMC proliferation. As shown in Figure 2A and B, up to 5% concentrations of either VS or QYGS alone did not influence the proliferation of HMCs. When HMCs were pre-incubated with various doses of VS or QYGS (1.25%, 2.5%, and 5%) for 1 h prior to treatment with 10^−4^ mmol/L of Ang II, cell proliferation was significantly decreased in a dose-dependent manner, thereby indicating that both VS and QYGS possess a potent inhibitory activity against Ang II-induced HMC proliferation (Figure 2C and D).

### Inhibitory effects of QYGS on Ang II-induced ROS production in HMCs

**Figure 3:**
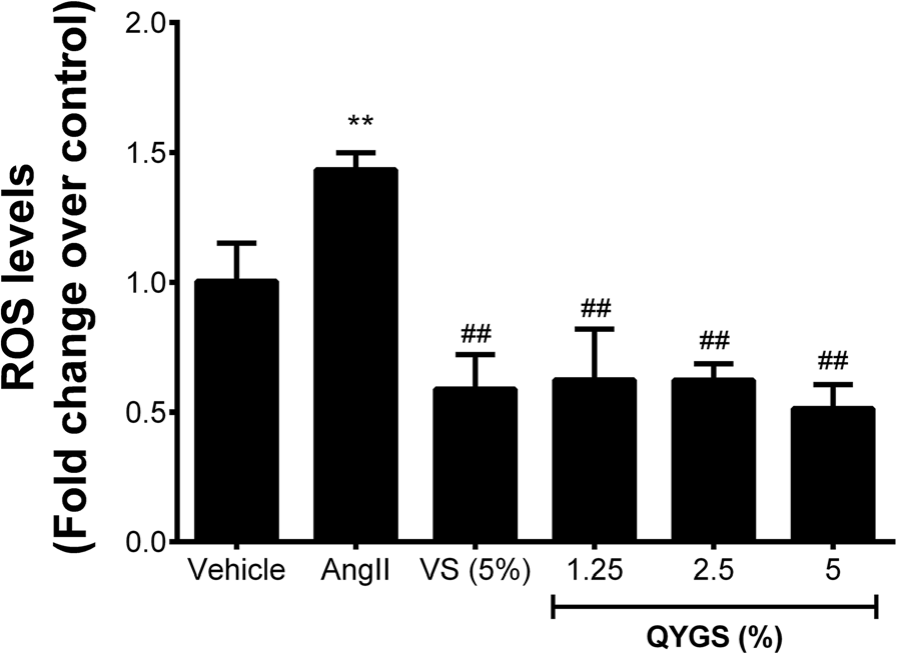
**Effects of QYGS on Ang II-induced ROS production in HMCs assessed by DCFH-DA assay.** Data are expressed as means ± SEM (n = 3). **P < 0.01 as compared with the vehicle group. ^##^ *P* < 0.01 as compared with the Ang II model group.

Figure 3 shows the inhibitory effects of QYGS on Ang II-induced ROS production in HMCs. Using the DCFH-DA method, we found a significant production of ROS when HMCs were treated with 10^−4^ mmol/L of Ang II for 24 h (Figure 3). However, ROS levels were considerably decreased after pre-incubation with 5% VS or with 1.25%, 2.5%, or 5% QYGS for 1 h prior to Ang II treatment. In addition, a dose-dependent effect was observed for QYGS pre-incubation. Collectively, these findings demonstrate that QYGS is able to suppress Ang II-induced ROS generation in HMCs.

### Effects of QYGS on mRNA and protein expression of NOX4, p22^*phox*^, and Rac1/GTP-Rac1 in Ang II-treated HMCs

As previously reported, Ang II could induce activation of NOX4 and Rac1 in mesangial cells. NOX4 forms a heterodimer with p22^*phox*^ subunits to facilitate ROS generation, while Rac1 uses Nox4-derived ROS as signal transducers to stimulate downstream signaling cascades [19]. In combining this information with the results shown in Figure 3, we assumed that the inhibitory effects of QYGS on Ang II-induced ROS production in HMCs may be mediated by a NOX4-dependent pathway.

**Figure 4:**
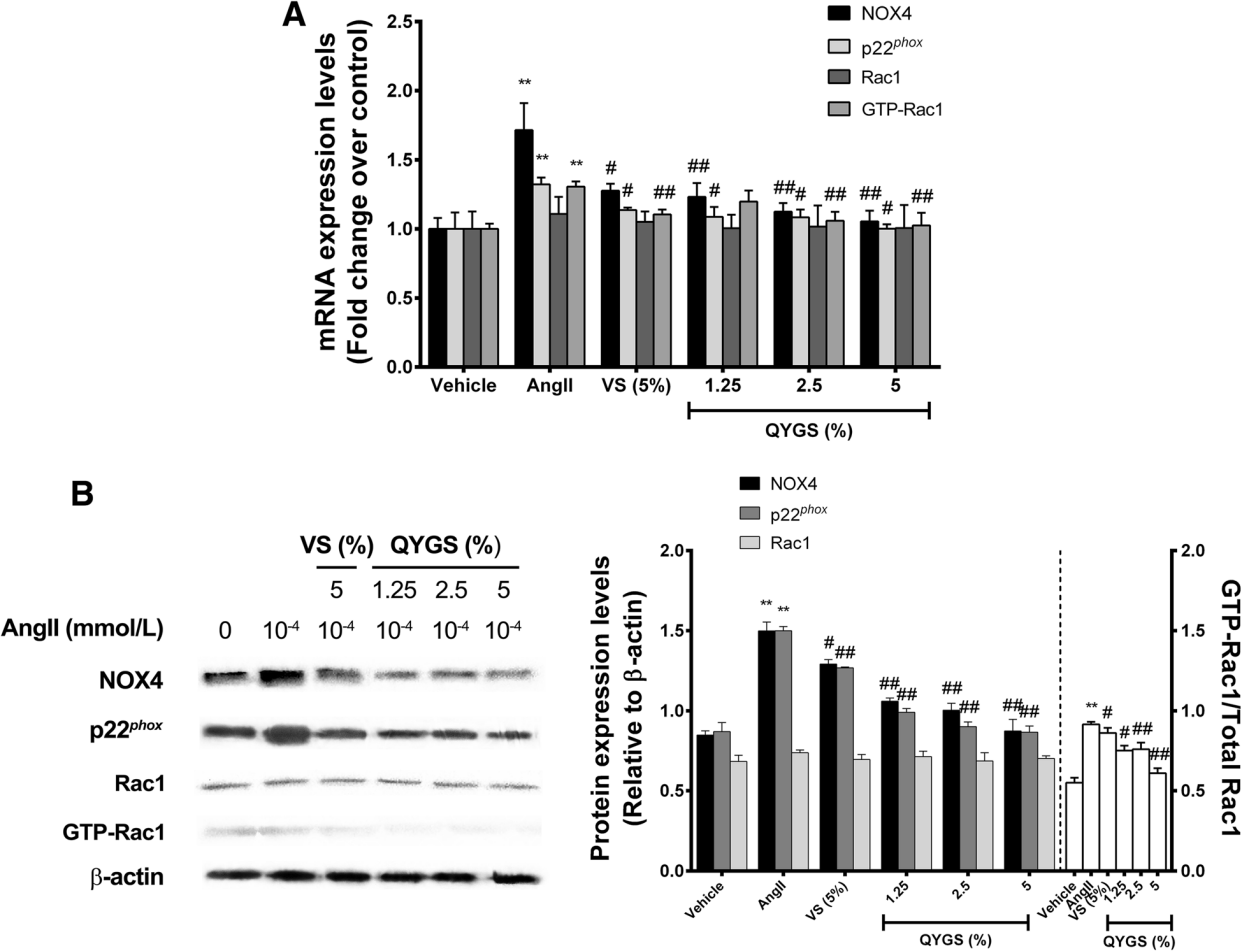
**QYGS counteracts Ang II-induced up-regulation of NOX4, p22** ^***phox***^ **, and GTP-Rac1 in HMCs.** Cells were pre-incubated with 5% VS or with 1.25%, 2.5%, or 5% QYGS for 1 h prior to treatment with 10^−4^ mmol/L of Ang II. RT-PCR and Western blot were carried out to detect mRNA **(A)** and protein levels **(B)**. Data are expressed as means ± SEM (n = 3). **P < 0.01 as ompared with the vehicle group. ^#^ *P* < 0.05, ^##^ *P* < 0.01 as compared with the Ang II model group.

In the current study, we found that treatment with 10^−4^ mmol/L of Ang II could induce a significant increase in mRNA and protein levels of NOX4, p22^*phox*^, and activated GTP-Rac1 (Figure 4A and B). Nevertheless, these effects could be counteracted by 5% VS or by QYGS in a dose-dependent manner (from 1.25% to 5%). For Rac1, however, no notable difference was observed in its mRNA and protein expression among all the groups. Taken together, these findings provide supportive evidence that QYGS is able to suppress the activation of the NOX4-dependent pathway in Ang II-treated HMCs, which may be responsible for attenuating Ang II-induced ROS production.

### Effects of QYGS on expression of TNF-α, NF-κB p65, and IL-6 in Ang II-treated HMCs

**Figure 5:**
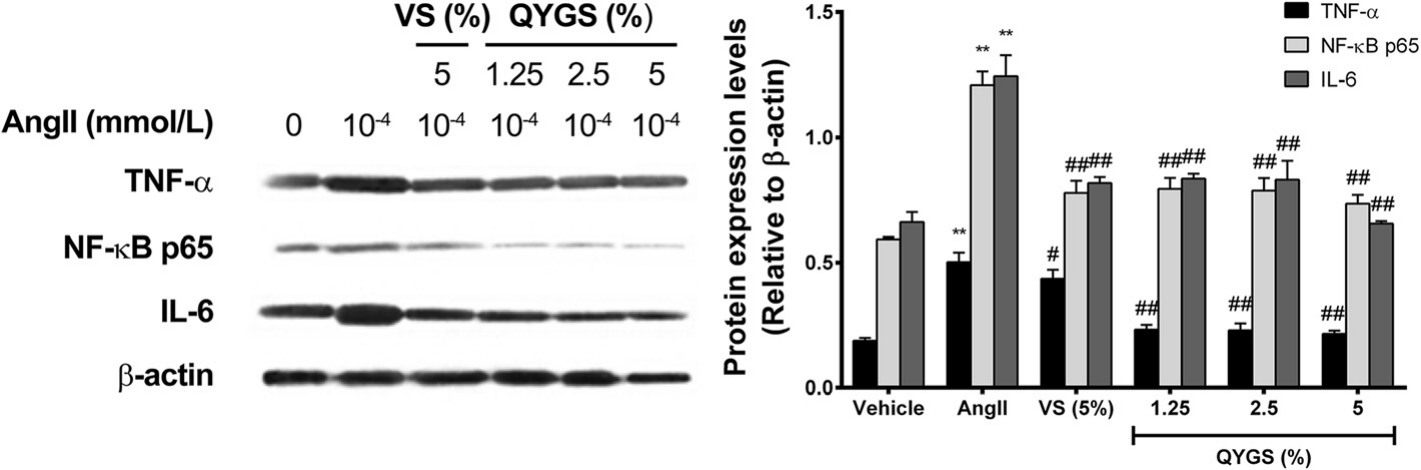
**Effects of QYGS on protein expression of TNF-α, NF-κB P65, and IL-6 in Ang II-treated HMCs as assessed by Western blot.** Data are presented as means ± SEM (n = 3). **P < 0.01 as compared with the vehicle group. ^#^ *P* < 0.05, ^##^ *P* < 0.01 as compared with the Ang II model group.

In taking into consideration that ROS-induced oxidative stress plays an essential role in activating inflammatory pathways in pro-inflammatory responses [20], we investigated the effects of QYGS on the expression of inflammatory markers, including TNF-α, NF-κB p65, and IL-6. As shown in Figure 5, treatment with 10^−4^ mmol/L of Ang II induced a significant increase in the protein expression of these inflammatory markers. After incubation with 5% VS or with 1.25%, 2.5%, or 5% QYGS for 1 h prior to Ang II treatment, the effects of Ang II on the expression of the three marker proteins were significantly counteracted. These results indicate that QYGS is able to prevent Ang II-induced pro-inflammatory responses in HMCs.

### Silencing RNA against NOX4 in Ang II-treated HMCs

**Figure 6:**
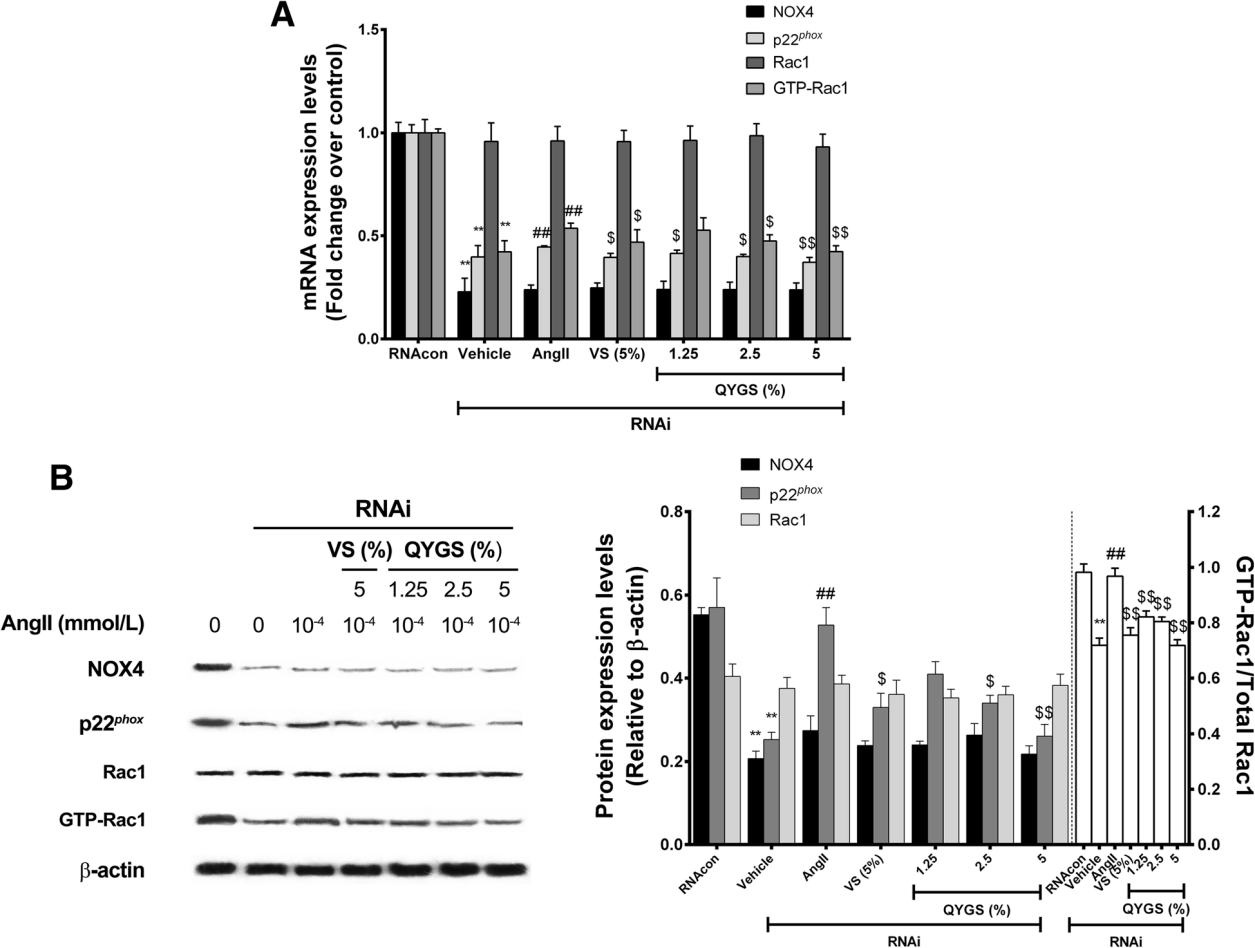
**QYGS suppresses Ang II-induced up-regulation of NOX4, p22** ^***phox***^ **, and GTP-Rac1 in NOX4 siRNA-transfected HMCs.** RT-PCR **(A)** and Western blot analysis **(B)** results of the effects of QYGS on NOX4, p22^*phox*^, and GTP-Rac1 upon transfection with non-targeting siRNA or NOX4 siRNA. Data are presented as means ± SEM (n = 3). **P < 0.01 as compared with the RNAcon group. ^#^ *P* < 0.05, ^##^ *P* < 0.01 as compared with the vehicle group. ^$^ *P* < 0.05, ^$$^ *P* < 0.01 as compared with the Ang II model group.

For further analysis, we employed siRNA to silence NOX4 expression in HMCs. After transfection with NOX4 siRNA for 24 h, RT-PCR showed that the mRNA expression of NOX4, p22^*phox*^, and GTP-Rac1 in HMCs had decreased by 72.7%, 69.3%, and 61.8%, respectively, which was statistically significant as compared with the RNAcon group (Figure 6A). These results demonstrate that the transfection of NOX4 siRNA was successful with a silencing efficiency of over 70%. After cells transfected with NOX4 siRNA were treated with 10^−4^ mmol/L of Ang II for 24 h, we found that mRNA expression levels of p22^*phox*^ and GTP-Rac1 were still significantly increased as compared with the vehicle group, indicating that the NADPH-oxidase activity was not completely inhibited upon siRNA knockdown of NOX4 and that other NADPH-oxidases may be activated by Ang II, thereby leading to up-regulation of p22^*phox*^ and GTP-Rac1 expression. When cells were pre-incubated with 5% VS or with 1.25%, 2.5%, or 5% QYGS for 1 h followed by Ang II stimulation, the expression levels of p22^*phox*^ and GTP-Rac1 were significantly decreased; and a dose-dependent effect was noted for QYGS pre-incubation. For Rac1, however, no significant difference was observed in mRNA levels among the groups overall. Similar results showing decreased protein levels of p22^*phox*^ and GTP-Rac1 in QYGS-pretreated HMCs were also obtained by Western blot analysis (Figure 6B).

**Figure 7:**
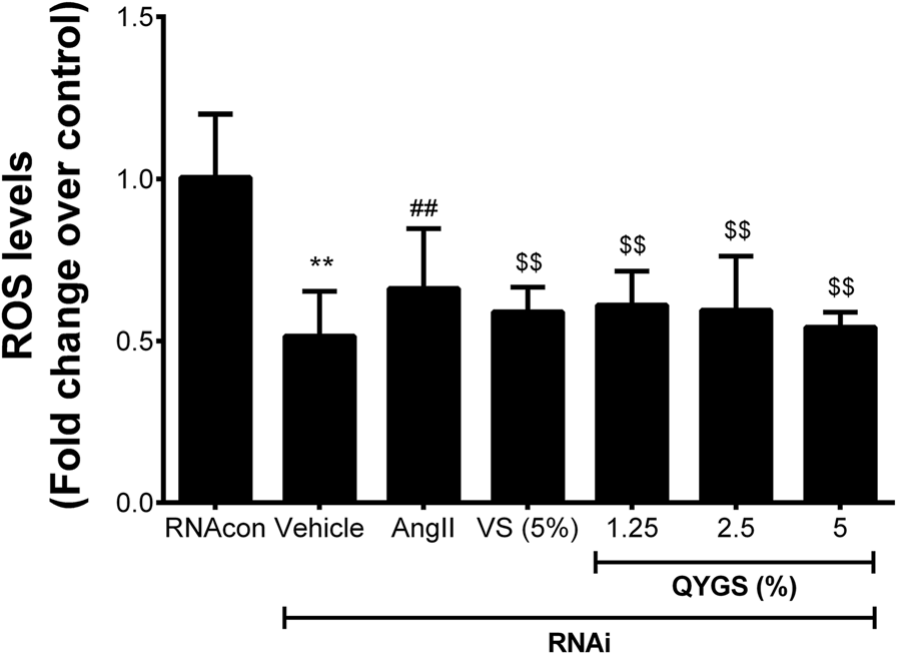
**Effects of QYGS on Ang II-induced ROS production in NOX4 siRNA-transfected HMCs as assessed by DCFH-DA assay.** Data are expressed as means ± SEM (n = 3). **P < 0.01 as compared with the RNAcon group. ^##^ *P* < 0.01 as compared with the vehicle group. ^$$^ *P* < 0.01 as compared with the Ang II model group.

Furthermore, we examined ROS production in siRNA-transfected HMCs, the results of which are shown in Figure 7. After transfection with NOX4 siRNA for 24 h, DCFH-DA assay showed that the ROS production in HMCs was significantly reduced when compared with the RNAcon group. However, treatment with 10^−4^ mmol/L of Ang II was able to induce a considerable increase in ROS production in siRNA-transfected cells. When cells were pre-treated with 5% VS or various doses of QYGS, this inductive effect of Ang II could be significantly inhibited, further demonstrating the ability of QYGS to inhibit Ang II-induced ROS generation in HMCs.

**Figure 8:**
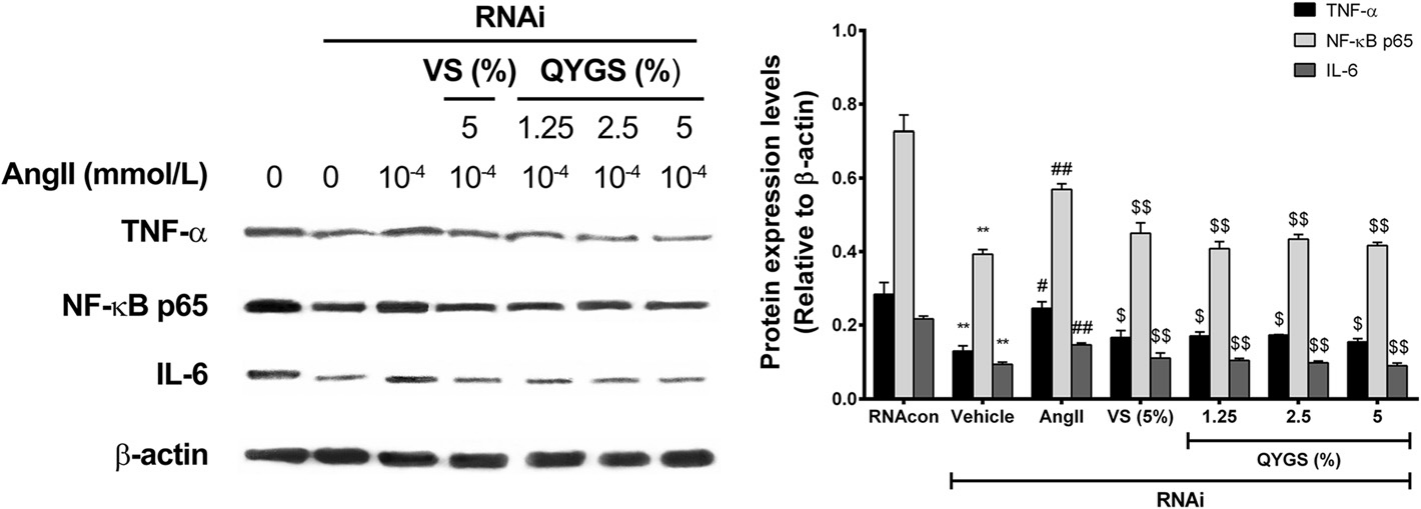
**Effects of QYGS on Ang II-mediated up-regulation TNF-α, NF-κB P65, and IL-6 in NOX4 siRNA-transfected HMCs as assessed by Western blot analysis.** Data are presented as means ± SEM (n = 3). **P < 0.01 as compared with the RNAcon group. ^#^ *P* < 0.05, ^##^ *P* < 0.01 as compared with the vehicle group. ^$$^ *P* < 0.01 as compared with the Ang II model group.

In order to further verify the effects of QYGS on suppressing Ang II-induced pro-inflammatory responses, we detected the protein levels of TNF-α, NF-κB p65, and IL-6 in HMCs after siRNA transfection. As shown in Figure 8, siRNA knockdown of NOX4 led to a considerable decrease in expression of the three inflammatory marker proteins as compared to the RNAcon group. When cells were incubated with Ang II, incubation caused a notable increase in the protein levels of all inflammatory markers. However, this effect was counteracted by pre-treatment with 5% VS or various doses of QYGS.

## Discussion

In the current study, we report for the first time that QYGS has inhibitory effects on Ang II-induced proliferation in HMCs. Furthermore, we found that QYGS is able to attenuate Ang II-induced ROS generation and pro-inflammatory responses in cells. These effects may be mediated by an NOX4-dependent pathway.

Renal vascular remodeling, hyperplasia of the glomerular mesangium, and renal fibrosis have been considered to be the main pathogenic factors behind renal damage in hypertension [21]. Hyperplasia of the glomerular mesangium is characterized by the proliferation of glomerular mesangial cells and excessive ECM accumulation [21,22]; while the key feature of renal fibrosis is the accumulation of myofibroblasts and ECM deposition [23]. Therefore, both the proliferation of glomerular mesangial cells and ECM deposition have been implicated as two key events in the progression of renal damage [24]. RAS, and particularly Ang II, is well known to be involved in a wide variety of cardiovascular pathologies including hypertension [2]. Researchers have demonstrated that Ang II is able to stimulate glomerular mesangial cell proliferation and ECM deposition, consequently causing glomerular sclerosis and additional renal damage [3]. It is therefore widely accepted that inhibition of Ang II-induced glomerular mesangial cell proliferation and ECM deposition should be an effective approach to preventing the development or progression of renal damage in hypertension [25,26]. In the current study, we found that pre-treatment with various doses of QYGS (from 1.25% to 5%), like pretreatment with 5% VS, could effectively inhibit Ang II-induced HMC proliferation. This effect may be due to the antioxidant and anti-inflammatory properties of the active components of QYYYG.

QYYYG contains six Chinese medical herbs, of which *Polygoni Multiflori Radix* is the major active component and has been indicated for the treatment of hypertension and hypertension-related renal diseases in Chinese Traditional Medicine for a number of decades. In recent years, numerous studies have proven that 2,3,5,4′-tetrahydroxystilbene-2-O-β-D-glucopyranoside (THSG) is the main active substance in *Polygoni Multiflori Radix*, which produces potent activity against the oxidative stress that follows acute inflammation [27-29]. In our study, although QYGS was used instead of QYYYG as intervention in HMCs, HPLC analysis showed that there was a high content of THSG in QYGS (data not shown). Furthermore, researchers have reported that some active substances extracted from *Corni Fructus*, such as 7-O-galloyl-D-sedoheptulose and loganin, have potent protective effects against diabetes- or glycerol-induced renal injury through elimination of ROS and inhibition of inflammation [30,31]. Therefore, we assume that the antioxidant and anti-inflammatory activity of these compounds may be involved in the inhibitory effect of QYGS on Ang II-induced HMC proliferation.

Recently, many studies have confirmed that oxidative stress and inflammation are important hallmarks of renal damage in hypertension [32,33]. As the key component of RAS, Ang II induces oxidative stress and inflammation by activating the Ang II type 1 (AT1) receptor [34]. Under pathological conditions, inflammatory changes induced by Ang II can lead to proliferation of mesangial cells and loss of the mesangial matrix, followed by excessive production of ECM and mesangial expansion [2]. Growing evidence suggests that these effects of Ang II are mainly mediated via activation of NOXs [7]. Researchers have demonstrated that NOXs are widely expressed in renal tissue [35]. Currently, at least four NOXs (NOX1, NOX2, NOX4, and NOX5) have been identified, of which NOX4 shows the highest expression level [36]. Recent studies have indicated that NOX4 is associated with p22^*phox*^ and serves as an important source of ROS; while Rac1 uses Nox4-derived ROS to elicit downstream signaling pathways [36,37]. Gorin et al. have provided definitive evidence that Ang II is able to induce activation of NOX4 and Rac1 in mesangial cells [19]. In line with their findings, we also found that incubation with 10^−4^ mmol/L of Ang II could not only induce a significant increase in ROS production in HMCs, but could also considerably enhance the mRNA and protein levels of NOX4, p22^*phox*^, and activated GTP-Rac1 as compared to the vehicle group. When cells were pre-treated with QYGS, we found that QYGS was able to decrease ROS production and down-regulate mRNA and protein expression of NOX4, p22^*phox*^, and GTP-Rac1 in Ang II-treated HMCs. These results suggest that these effects of QYGS may be mediated through a NOX4-dependent pathway. Nevertheless, when NOX4 was silenced by siRNA in HMCs, Ang II was able to facilitate ROS production and expression of the aforementioned molecules to at least some extent; but these effects were still counteracted by intervention with QYGS. According to previous literature, Ang II is also functionally associated with NOX1, NOX2, and NOX5 [36]. Therefore, these findings imply that other NOXs might also be involved in the antioxidant potency of QYGS. However, extensive studies are required to confirm this.

Numerous studies have revealed that ROS production upon Ang II-induced NOX activation promotes downstream inflammatory responses, which are critical for the development and progression of renal damage in hypertension [38,39]. The signaling pathways involved in this process include NF-κB, STAT, and MAPK pathways, which can be activated by ROS to regulate the gene expression of numerous cytokines, as well as adhesion and chemoattractant molecules [40]. In this study, we found that QYGS is able to inhibit the Ang II-induced protein expression of TNF-α, NF-κB p65, and IL-6 in HMCs. This finding demonstrates the inhibitory effect of QYGS on Ang II-induced pro-inflammatory responses, which is assumed to be related to the negative impact of QYGS on Ang II/NOX pathway. However, a thorough understanding of how the active ingredients in QYGS, such as THSG, exert their signaling functions still awaits further research.

One possible limitation to this study should be noted. According to Cao et al., rat and human exhibit different expression levels and patterns for metabolizing enzymes in the intestine [41]. Hence, it is reasonable to assume that the metabolism of QYYYG in rat may be different with that in human, and that the use of QYGS might introduce a bias into the results of this study. Therefore, further studies using QYYYG-containing human serum are required to validate the present findings.

## Conclusions

In summary, the current study shows that QYGS is able to inhibit cell proliferation, ROS production, and inflammation in Ang-II-treated HMCs. Our data suggest that an NOX4-dependent pathway plays an important role in regulating the inhibitory effect of QYGS. These findings provide new insights into the molecular mechanisms of QYYYG in the treatment of renal damage in hypertension.

## Abbreviations

Ang II: Angiotensin II
ARBs: Angiotensin receptor blockers
DCHF-DA: 2′,7′-dichlorodihydrofluorescein diacetate
DMSO: Dimethyl sulfoxide
ECM: Extracellular matrix
GTP-Rac1: Activated Ras-related C3 botulinum toxin substrate 1
HMCs: Human mesangial cells
IL-6: Interleukin 6
MTT: 3-(4,5-dimethyl-2-thiazolyl)-2,5-diphenyl tetrazolium bromide
NF-κB p65: Nuclear factor-κB p65
NOX: Nicotinamide adenine dinucleotide phosphate (NAPDH)-oxidase
QYGS: QYYYG-containing serum
QYYYG: Qian Yang Yu Yin Granule
RAS: The renin-angiotensin system
ROS: Reactive oxygen species
siRNA: Small interfering RNA
TNF-α: Tumor necrosis factor-α
VS: Valsartan-containing serum
